# A Phenylbutenoid Dimer, *cis*-3-(3′,4′-Dimethoxyphenyl)-4-[(*E*)-3′′′,4′′′-Dimethoxystyryl] Cyclohex-1-ene, Exhibits Apoptogenic Properties in T-Acute Lymphoblastic Leukemia Cells via Induction of p53-Independent Mitochondrial Signalling Pathway

**DOI:** 10.1155/2013/939810

**Published:** 2013-02-28

**Authors:** Theebaa Anasamy, Ahmad Bustamam Abdul, Mohd Aspollah Sukari, Siddig Ibrahim Abdelwahab, Syam Mohan, Behnam Kamalidehghan, Mohd Zulkhairi Azid, Nabilah Muhammad Nadzri, A. Reenaa Joys Andas, Ng Kuan Beng, A. Hamid A. Hadi, Heshu Sulaiman Rahman

**Affiliations:** ^1^UPM-MAKNA Cancer Research Laboratory, Institute of Bioscience, University Putra Malaysia, 43400 Serdang, Selangor, Malaysia; ^2^Department of Chemistry, Faculty of Science, University Putra Malaysia, 43400 Serdang, Selangor, Malaysia; ^3^Department of Pharmacy, Faculty of Medicine, University of Malaya, 50603 Kuala Lumpur, Malaysia; ^4^Medical Research Center, Faculty of Medicine, Jazan University, Jazan, P.O. Box 114, Saudi Arabia; ^5^Faculty of Science, University of Malaya, 50603 Kuala Lumpur, Malaysia; ^6^Department of Microbiology and Pathology, Faculty of Veterinary Medicine, University Putra Malaysia, 43400 UPM Serdang, Selangor, Malaysia

## Abstract

The current study was designed to evaluate the *in vitro* cytotoxicity effect of a phenylbutenoid dimer, *cis*-3-(3′,4′-dimethoxyphenyl)-4-[(*E*)-3^**‴**^,4^**‴**^-dimethoxystyryl]cyclohex-1-ene (ZC-B11) isolated from the rhizome of *Zingiber cassumunar* on various cancer cell line, and normal human blood mononuclear cells, and to further investigate the involvement of apoptosis-related proteins that leads, to the probable pathway in which apoptosis is triggered. Cytotoxicity test using MTT assay showed selective inhibition of ZC-B11 towards T-acute lymphoblastic leukemia cells, CEMss, with an IC_50_ value of 7.11 ± 0.240 **μ**g/mL, which did not reveal cytotoxic effects towards normal human blood mononuclear cells (IC_50_ > 50 **μ**g/mL). Morphology assessments demonstrated distinctive morphological changes corresponding to a typical apoptosis. ZC-B11 also arrested cell cycle progression at S phase and causes DNA fragmentation in CEMss cells. Decline of mitochondrial membrane potential was also determined qualitatively. In the apoptosis-related protein determination, ZC-B11 was found to significantly upregulate Bax, caspase 3/7, caspase 9, cytochrome c, and SMAC and downregulate Bcl-2, HSP70, and XIAP, but did not affect caspase 8, p53, and BID. These results demonstrated for the first time the apoptogenic property of ZC-B11 on CEMss cell line, leading to the programmed cell death via intrinsic mitochondrial pathway of apoptosis induction.

## 1. Introduction

Plants, in particular have been used by mankind as a source of medicine since the times of yore. Evidence on plants with healing properties exists about 5000 years ago, which had been documented since Sumerian civilization [1]. In about 3000 to 1500 years ago, curative treatments using plant-based substances had been practiced and increased widely in ancient Greece, followed by useful knowledge of plants having therapeutic values in China, India, and Tibet, 1000 to 2000 years ago [2]. These pieces ancient knowledge were brought down from one generation to another and with the advancement of science and technology, plant-derived drugs have made massive contributions in various clinical conditions and had provided important leads against various pharmacological targets including cancer [3]. 

*Zingiber cassumunar *(family: Zingiberaceae) is commonly known as “Plai” in Thailand and “Bonglai” in Malaysia. It has long been used in traditional medicine in Thailand, being the prime ingredient in massage oil to relieve muscle pain. In Northeast India, oral consumption of the rhizome paste of *Z. cassumunar* was reported to treat dyspepsia and stomach bloating [4, 5] whilst in Malaysia, it is used for postpartum medication. 

**Figure 1:**
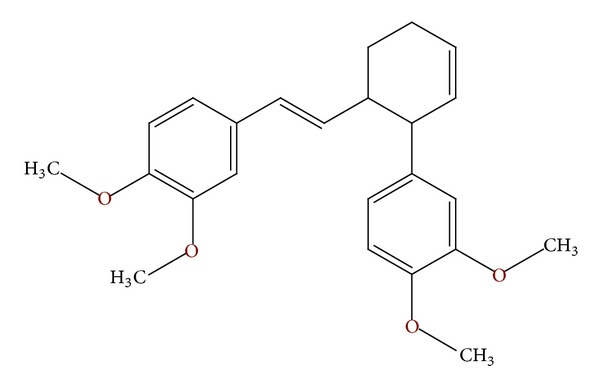
The chemical structure of ZC-B11.

*cis*-3-(3′,4′-Dimethoxyphenyl)-4-[(*E*)-3′′′,4′′′-dimethoxystyryl]cyclohex-1-ene (ZC-B11) is a phenylbutenoid dimer isolated from the rhizomes of *Z. cassumunar* (Figure 1). To date, there have been no reported studies on ZC-B11 isolated from the rhizome of *Z. cassumunar* having antileukemic activity. Therefore, the current study was conducted to investigate the *in vitro* antileukemic properties of this compound to substantiate its anticancer activity.

## 2. Materials and Methods

### 2.1. Compound Isolation and Purification

The rhizomes of *Z. cassumunar* were collected from Jogjakarta, Indonesia, in the year 2007. Voucher specimen was deposited in Herbarium of Faculty of Pharmacy, Gajah Mada University, Jogjakarta. Briefly, the finely ground rhizomes of *Z. cassumunar* (~700 g) were soaked in petroleum ether for 72 hours at room temperature. The extraction was repeated 3 times to remove the nonpolar organic compounds, waxes, and fats. Extraction was continued with chloroform, ethyl acetate, and methanol. The solvents were removed under reduced pressure and crude extracts were obtained. Column chromatography over silica gel using a stepwise gradient elution system was utilized to fractionate the petroleum ether extract (25 g). The isolation of the crude extract yielded 64 fractions. Fractions 12-13 showed similar pattern on TLC and were later combined. Purification of this fraction was done by column chromatography using mixture of hexane and ethyl acetate as eluent. Subfraction B11 was collected from hexane: EtOAc (8 : 2) and was further washed with hexane and methanol to give white solid, *cis*-3-(3′,4′-dimethoxyphenyl)-4-[(*E*)-3′′′,4′′′-dimethoxystyryl]cyclohex-1-ene. The molecular weight of this compound is 380.199 g/mol with molecular formula C_24_H_28_O_4_ and melting point 91°C-92°C [6]. The compound was sent for infrared (IR) and nuclear magnetic resonance (NMR) analyses at the laboratory of spectroscopic analysis, Faculty of Science, UPM. ^1^H NMR spectra were recorded on NMR: Bruker Avance 400 spectrometer apparatus, *¹*³C NMR spectra were reported at Ac 150 MHz instrument, and Electron Impact Mass Spectra (EI-MS) were recorded on Finnigan MAT 31 mass spectrometer with a MATSPECO data system. EI-MS analysis indicated the presence of molecular ion peak at m/z 380 which corresponded to the molecular formula of C_24_H_28_O_4_. The spectral data were found to be in good agreement with the published data [6]. No previous studies have been reported on this compound except for its phytochemical structure determination and physicochemical characterization.

*cis*-3-(3′,4′-Dimethoxyphenyl)-4-[(*E*)-3′′′,4′′′-dimethoxystyryl]cyclohex-1-ene: IR *υ*_max⁡_ (cm^−1^, UATR): 3017 (=C–H), 2927 (C–O), 1589 (C=C), 1509, 1458, 1233, 1138, 1019, 853, 785, 680, 614; ^1^H NMR (400 MHz, CDCl_3_): *δ* 6.80 (1H, *d*, *J* = 8.0 Hz, H-5′), 6.76 (3H, *br.s*, H-6′, H-6′′′, H-5′′′), 6.73 (1H, *s*, H-2′′′), 6.70 (1H, *s*, H-2′), 6.26 (1H, *d*, *J* = 16.0 Hz, H-7′′), 5.81 (1H, *dt*, *J* = 10.1, 3.6 Hz, H-1), 5.99 (1H, *d*, *J* = 10.1 Hz, H-2), 5.59 (1H, *dd*, *J* = 16.0, 9.0 Hz, H-8′′), 3.86 (3H, *s*), 3.75 (3H, *s*), 3.86 (3H, *s*), 3.83 (3H, *s*), 3.51 (1H, *br. s*, H-3), 2.72 (1H, *m*, H-4), 2.22 (2H, *m*, H-6), 1.68 (2H, *t*, *J* = 7.0 Hz, H-5); ^13^C NMR (100 MHz, CDCl_3_): *δ* 148.9 (C-3′′′-OCH_3_), 148.2 (C-4′′′—OCH_3_), 148.0 (C-3′—OCH_3_), 147.5 (C-4′—OCH_3_), 133.8 (C-1′), 132.4 (C-8′′), 131.0 (C-1′′′), 128.0 (C-2), 128.5 (C-7′′), 129.0 (C-1), 121.9 (C-6′), 118.7 (C-6′′′), 113.7 (C-2′), 111.1 (C-5′′′), 110.3 (C-5′), 108.9 (C-2′′′), 45.8 (C-3), 42.6 (C-4), 24.8 (C-6), 24.3 (C-5); MS m/z (% intensity): 380 (M^+^, 15), 300 (2), 229 (2), 190 (100), 175 (17), 159 (80), 144 (17).

### 2.2. Cell Lines and Reagents

All cancer cell lines were obtained from American Type Culture Collection (ATCC). RPMI 1640 and Fetal Bovine Serum (FBS) were purchased from PAA (Germany). DMSO, penicillin, and streptomycin solution were purchased from Sigma (St. Louis, MO, USA). MTT was purchased from Amresco (USA). Quantum PBL media was purchased from PAA (Austria). Phosphate buffer saline was obtained from Invitrogen (Carlsbad, USA). All other chemicals and reagents used were of HPLC grade.

### 2.3. Cell Culture

Cancer cells were cultured in RPMI 1640 medium supplemented with 10% FBS and 1% 100 unit/mL penicillin and 100 *μ*g/mL streptomycin. Cultures were maintained at 37°C in a humidified 5% CO_2_ incubator. Experiments were performed at concentration of 200,000 cells/mL.

### 2.4. Cell Viability Assay on Cancer Cells

 T-Acute lymphoblastic leukemia (CEMss), hepatocellular carcinoma (HepG2), human breast adenocarcinoma (MCF-7), human breast carcinoma (MDA-MB-231), and cervical carcinoma (HeLa) were used in this study. Cell suspension of each cell line was plated out into 96-well plates and treated with different concentrations (1.563, 3.125, 6.25, 12.5, 25, and 50 *μ*g/mL) of ZC-B11. Control wells included vehicle-treated cells exposed to 0.1% (w/v) DMSO. After 68 h incubation, MTT (5 mg/mL) was added to each well and the plate was incubated for further 4 h. Supernatants were removed before adding 100 *μ*L DMSO to solubilise the formazan crystals formed. Absorbance was read at wavelength of 595 nm using a microplate reader (Tecan Sunrise Basic, Groedig, Austria). Assay was performed in triplicates to calculate IC_50_ values (concentration which inhibits 50% of cellular growth). CEMss cells were also treated with 5-fluorouracil used as positive control.

### 2.5. Cytotoxicity of ZC-B11 on Human Blood Mononuclear Cells

The ability of ZC-B11 to act selectively on cancer cells especially leukemia was evaluated by comparing the cytotoxicity of this compound towards human blood mononuclear cells. Briefly, blood was collected into the cell preparation tube containing sodium citrate (BD Vacutainer, NJ, USA). After collection, tube was stood upright for 20 min at room temperature to allow it to equilibrate and later centrifuged at 1200 ×g for 20 min. Mononuclear cells and platelets underneath the plasma layer were collected using a pipette and transferred into 15 mL centrifuge tube. Cells were washed twice with PBS and cultured in complete Quantum PBL media with phytohemagglutinin (PAA, Pasching, Austria) containing 10% FBS supplemented with 100 U/mL penicillin and 100 *μ*g/mL streptomycin at 37°C in 5% CO_2_ atmosphere. Human blood mononuclear cells were treated at various concentrations of ZC-B11 in triplicates and cell viability was measured using MTT assay after 72 h of incubation.

### 2.6. Microscopic Observation of Cellular Morphology Using Phase-Contrast-Inverted Microscopy

This investigation examines morphologically if cell death induction is implicated in ZC-B11-treated CEMss cell. CEMss cells were exposed to 7.11 *μ*g/mL (IC_50_) of ZC-BII for 24, 48, and 72 h. Morphological appearances of treated CEMss cells were compared with untreated control observed under normal phase contrast inverted microscope. Cells were identified as undergoing apoptosis cell death if they display condensed nuclear, fragmented nuclei, and/or blebbing.

### 2.7. Confocal Microscopy (Acridine Orange, AO and Propidium Iodide, and PI Double Staining)

Morphological assessments of treated and untreated CEMss cells were done using a double-fluorescent dye staining method. Briefly, CEMss cells were treated with IC_50_ concentration of ZC-BII for 24, 48, and 72 h. After the treatment period, cells were washed twice using PBS to remove the remaining media. Ten *μ*L (10 *μ*L) of fluorescent dyes, (AO/PI) containing AO (10 *μ*g/mL) and PI (10 *μ*g/mL), was added into the cellular pellet in equal volumes. Freshly stained cell suspension was then dropped onto glass slides and covered by coverslip. Slides were observed under confocal microscope within 30 min before the fluorescence colour starts to fade. The criteria for identification are as follows: (a) green intact nucleus, viable cells; (b) dense green areas of chromatin condensation in the nucleus, early apoptosis; (c) dense orange areas of chromatin condensation, late apoptosis; and (d) orange intact nucleus, secondary necrosis [7].

### 2.8. Phosphatidylserine Externalisation Study

Phosphatidylserine (PS) externalisation study was done using AnnexinV:FITCassay kit (AbD Serotec, USA). CEMss cells were treated with ZC-BII at IC_50_ concentration for 24, 48, and 72 h, while untreated cells were used as negative control. After the treatment period, the supernatant was discarded and cells were washed twice using PBS. Cells were resuspended in prediluted binding buffer in 1 : 4 ratio (50 mL binding buffer + 150 mL distilled water); later 5 *μ*L Annexin V:FITC was added into 195 *μ*L of the cell suspension, mixed well, and incubated for further 10 min in the dark, at room temperature. Cells were then washed and resuspended with 190 *μ*L of pre-diluted binding buffer followed by the addition of 10 *μ*L of the PI solution and analysed with flow cytometer (BD FACS Canto II, USA).

### 2.9. Cell Cycle Distribution Analysis

A time-dependent study of cell cycle distribution of CEMss cells treated with IC_50_ concentration ZC-BII was performed in triplicates. Untreated cells were used as negative control. After the incubation period (24, 48, and 72 h), cells were washed with PBS. To restore cell integrity, fixation of cell population for flow cytometry analysis was performed. Briefly, cell pellets were fixed with 90% cold ethanol by mixing 700 *μ*L of 90% cold ethanol and the resulting cell suspension was kept overnight at −20°C. The cell suspension was then centrifuged at 850 rpm for 10 minutes and the supernatant containing ethanol was removed. The cell pellet was washed using 2 mL PBS and later resuspended with 600 *μ*L of PBS + 10 mg/mL RNase + 1 mg/mL Propidium Iodide (PI). PI can bind to RNA molecule and thus, RNAse enzyme was added in order to allow PI to bind directly to DNA. The cells were then incubated between 30 min to 1 h at 37°C. Finally, cell cycle kinetics was examined using flow cytometer (BD FACS Canto II, USA). Fluorescence intensity of sub-G_0_/G_1_ cell fraction represents apoptotic cell population. 

### 2.10. DNA Fragmentation

DNA fragmentation was done using Suicide-Track DNA Ladder Isolation Kit (Calbiochem, Germany) according to the manufacturer's instructions. Briefly, CEMss cells were treated with ZC-BII at IC_50_ concentration for 48 h. After treatment, DNA extraction, which involves the separation of apoptotic DNA from high molecular weight chromatin, and DNA precipitation were performed according to the manufacturer's instructions. DNA gel electrophoresis was done by preparing agarose gel (1.2%). All DNA ladder samples (21 *μ*L each) including DNA markers (5 *μ*L) were transferred to clean centrifuge tubes and each sample was added with Novel Juice (GeneDirex, USA) (1-part Novel Juice with 5-part DNA sample). Novel Juice is a nonmutagenic fluorescent reagent (alternative to Ethidium Bromide) that produces instant visualization of DNA bands upon UV illumination of agarose gels. All samples were then loaded onto the gel and the gel was run at approximately 50 constant volts until the dye front is 1-2 cm from bottom of the gel. DNA was then visualized by transillumination with UV light (Biospectrum AC Chemi HR 40, UVP, Upland, CA, USA) and photographed.

### 2.11. Qualitative Analysis of Mitochondrial Membrane Potential

Mitochondrial membrane potential (MMP) was qualitatively analysed using Rhodamine 123 (Sigma, USA), a positive charged molecule that can accumulate in energized mitochondria, resulting in the decline of fluorescence intensity. Briefly, CEMss cells were treated with ZC-B11 compound at IC_50_ concentration for 12, 24, 48, and 72 h. Untreated cells serve as negative control. Cells in different treatment groups were adjusted to the same density, stained with 10 *μ*g/mL Rh123 in the dark followed by rinsing in PBS, and photographed under the fluorescent microscope (Olympus BX60F5, Japan).

### 2.12. Human Apoptosis Proteome Profiler Array

To determine the probable pathway of apoptosis induction mediated by ZC-B11 in CEMss cells, detection of several apoptosis-related markers was carried out using the Proteome Profiler Array (RayBio Human Apoptosis Antibody Array Kit, Raybiotech, USA) according to the manufacturer's instructions. Briefly, cells were treated with 7.11 *μ*g/mL of ZC-B11. Untreated cells were used as negative control. Three hundred micro gram proteins from each sample were incubated with the human apoptosis array overnight. The apoptosis array data were quantified by scanning the membrane on a Biospectrum AC ChemiHR 40 (UVP, Upland, CA, USA) and analysis of the array image was performed using image analysis software according to the manufacturer's instruction.

### 2.13. Bioluminescent Assay of Caspases 3/7, 8, and 9

Caspase 3/7, 8, and 9 activities of treated and untreated CEMss cells were measured using a Caspase-Glo assay kit (Promega Corp., Madison, WI, USA). Briefly, CEMss cells were seeded in a white-walled 96-well plate and treated with ZC-B11 at IC_50_ concentration for 24, 48, and 72 h. Untreated cells served as negative control. The Caspase-Glo 3/7, 8, and 9 reagents were mixed well and allowed to equilibrate at room temperature before starting the assay. The 96-well plate containing cells was removed from the incubator and allowed to equilibrate to room temperature. Then, 100 *μ*L of Caspase-Glo 3/7, 8, and 9 reagents were added into each well of the 96-well plate containing 100 *μ*L of blank (vehicle only), negative control cells or treated cells in culture medium. Contents inside the wells were gently mixed by using a plate shaker at 300–500 rpm for 30 seconds and incubated at room temperature between 30 min to 3 h. Luminescence of each sample was measured in a luminescence microplate reader (Infinite M200 PRO Tecan, Austria). Concisely, the proluminescent substrate containing the DEVD, LETD and LEHD (sequences are in a single-letter amino acid code) was cleaved by caspases 3/7, 8, and 9, respectively. After the caspase cleavage, a substrate for luciferase (aminoluciferin) is released, which eventually results in the luciferase reaction and the production of luminescent signal.

### 2.14. Western Blot

This analysis was used to investigate the expression of apoptosis-related proteins which included Bax, Bcl-2, and HSP70. CEMss cells were treated with ZC-B11 for 3, 6, 12, and 24 h. Untreated cells serve as negative control. Total proteins of cells were extracted with cell lysis buffer (50 mM Tris-HCL pH 8.0, 120 mM NaCl, 0.5% NP-40, 1 mM PMSF), and 40 *μ*g of protein extract was separated by 10% SDS PAGE and then transferred to a polyvinylidenedifluoride (PVDF) membrane (Bio-Rad, USA) using semidry transfer unit (Hoefer TE 70X, USA) blocked with 5% nonfat milk in TBS-Tween buffer (0.12 M Tris-base, 1.5 M NaCl, 0.1% Tween20) for 1 h at room temperature. The PVDF membrane was then incubated with appropriate primary antibody overnight at 4°C and then incubated with horseradish peroxidase-conjugated secondary antibody for 30 min at room temperature. The bound secondary antibody was detected using peroxidase-conjugated antirabbit antibody (1 : 10000) or antimouse antibody (1 : 10000), followed by its detection using colorimetric method. The following primary antibodies *β*-actin (1 : 10000), Bcl-2 (1 : 1000), Bax (1 : 1000), and HSP70 (1 : 1000) were purchased from Santa Cruz Biotechnology, Inc, CA, USA.

### 2.15. Statistical Analysis

Data were expressed as mean ± SD. Statistical analysis was performed using Student's *t*-test where *P* < 0.05 was considered to be statistically significant.

## 3. Results 

### 3.1. ZC-B11 Showed Potent Antiproliferative Effect on CEMss Cells but Does Not Inhibit Human Blood Mononuclear Cells

ZC-B11 was found to exert the most potent antiproliferative effect towards CEMss cells with IC_50_ value of 7.11 ± 0.24 *μ*g/mL followed by HepG2, MCF-7, MDA-MB-231, and HeLa cells with IC_50_ values of 17.65 ± 0.32 *μ*g/mL, 21.28 ± 0.25 *μ*g/mL, 32.38 ± 0.41 *μ*g/mL, and >50 *μ*g/mL, respectively, after 72 h incubation (Table 1). The antiproliferative activities of ZC-B11 on CEMss, HepG2, MCF-7, and MDA-MB-231 cell lines exhibited IC_50_ values below 30 *μ*g/mL. However, the lowest IC_50_ value of ZC-B11 on CEMss suggested preliminarily that ZC-B11 possesses high anticancer activity, which was to be suggested being useful against T-acute lymphoblastic leukemia. Thus, further experiments throughout this study were conducted using this cell line. 

The crucial objective of expanding molecularly targeted drugs is to improve the efficacy and selectivity of cancer treatment by exploiting the differences between cancer cells and normal cells [8]. Hence, the ability of ZC-B11 to act selectively on cancer cells especially leukemia was evaluated by comparing the cytotoxicity of this compound on human blood mononuclear cells. ZC-B11 did not produce any cytotoxic effect on human blood mononuclear cells up to the concentration of 50 *μ*g/mL. 5-Fluorouracil was used as positive control and it revealed an inhibitory effect towards T-acute lymphoblasticleukemia cell line (CEMss) with an IC_50_ value of 1.54 ± 0.035 *μ*g/mL. In respect to this, the sensitivity of CEMss to 5-fluorouracil correlated to the MTT assay result obtained for ZC-B11.

### 3.2. ZC-B11 Causes Morphological Changes Related to Apoptosis

**Figure 2:**
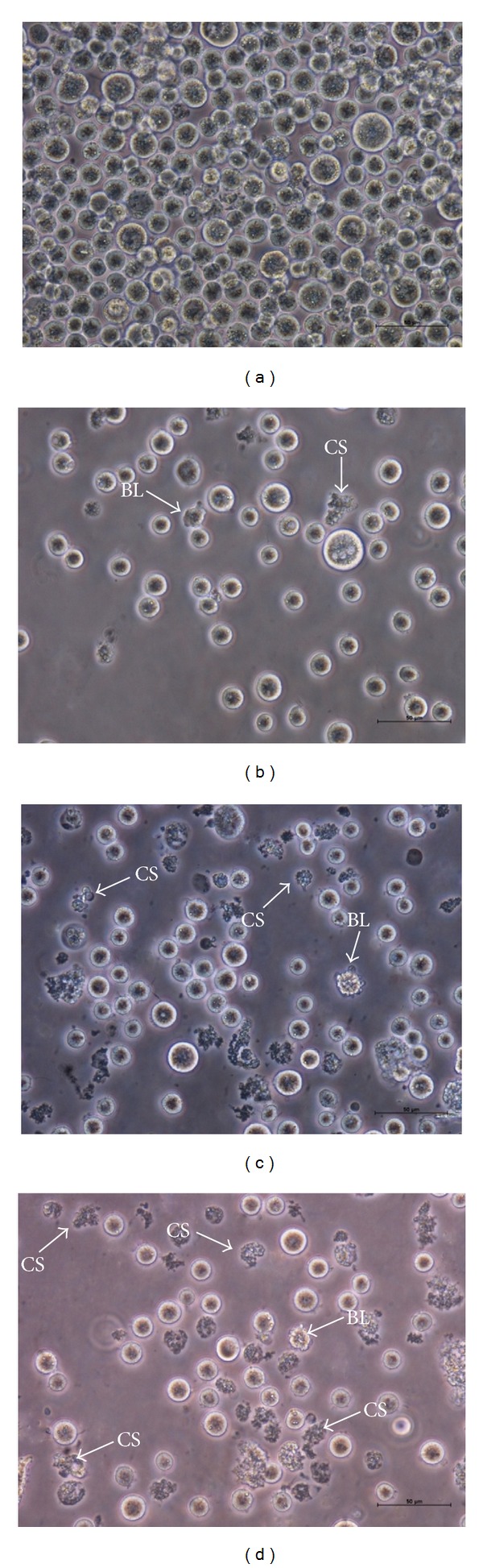
Normal phase contrast inverted micrograph of CEMss cells treated withZC-B11 (IC_50_) for 24, 48, and 72 h. (a) Control, (b) most of the cells exhibit normal morphology while some cells show cytoplasmic protrusions (24 h), (c) clear apoptogenic morphology such as blebbing and cell shrinkage observed (48 h), and (d) prominent growth inhibition, blebbing of the cell membrane, and shrinkage of cells observed (72 h). BL: blebbing of the cell membrane; CS: cell shrinkage (400x magnification).

**Figure 3:**
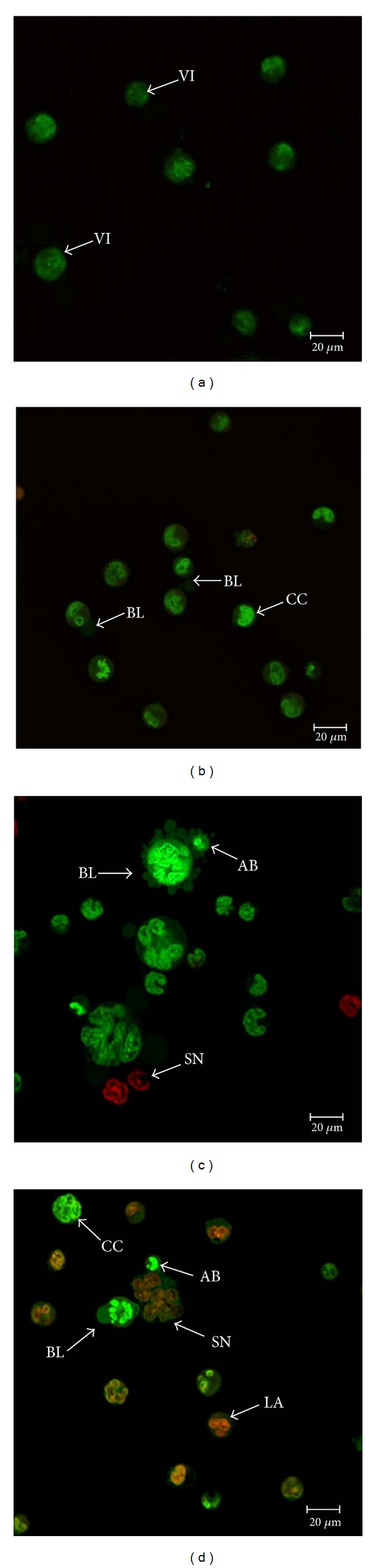
Confocal micrograph of acridine orange and propidium iodide double-stained CEMss cells after 24, 48, and 72 h treatment with ZC-B11 (IC_50_). (a) Control, (b) cells exhibit blebbing of the cell membrane and bright green nucleus showing condensation of chromatin (24 h), (c) blebbing was observed with some orange-coloured cells which denotes late apoptosis (48 h), and (d) more blebbing and late apoptosis, orange colour represents the hallmark of late apoptosis while red color represents secondary necrosis or dead cells (72 h). VI: viable cells; BL: blebbing of the cell membrane; CC: chromatin condensation; AB: apoptotic body; LA: late apoptosis; SN: secondary necrosis (400x magnification).

The effect of ZC-B11 on the morphology of CEMss cells was analyzed using normal phase contrast inverted microscopy at 24, 48, and 72 h incubation. Microscopic observation revealed morphological changes in CEMss cells treated with 7.11 *μ*g/mL of ZC-B11 in a time-dependent manner. ZC-B11-treated CEMss cells showed blebbing of the cell membrane and shrinkage of the cells. These apoptotic effects were found to be in a time dependent manner, which correlate well to the phenomenon of cell-death induction, considering that the number of blebs formation (cytoplasmic protrusion) increases as apoptosis progresses. After 24 h treatment, some cells remained healthy while some cells exhibited cytoplasmic protrusions (Figure 2(b)). The morphological changes were distinctively clear in treated CEMss cells after 48 and 72 h treatment with features of prominent growth inhibition, increased blebbing of the cell membrane, and shrinkage of cells (Figures 2(c) and 2(d)). In contrast, untreated cells showed typical nonadherent cell morphology and remained healthy and confluent throughout the treatment period (Figure 2(a)). In confocal microscopy aided with acridine orange and propidium iodide double staining, early apoptosis features such as blebbing and chromatin condensation were seen obviously in treated CEMss cells while untreated cells showed even distribution of the acridine orange stain as green intact nucleus (denotes healthy cells) with well-preserved morphology (Figure 3(a)). After 24 h exposure to ZC-B11, blebbing of the cell membrane and dense green nucleus which indicate nuclear chromatin condensation were noticeable (Figure 3(b)). The apoptotic characteristics of CEMss became more apparent at 48 h of treatment (Figure 3(c)) and prominent at 72 h (Figure 3(d)) where most of the cells exhibited dense green nucleus and blebbing compared to untreated and 24 h treatment. Both early apoptosis features (blebbing and chromatin condensation) and late phases of apoptosis, which specify presence of intense reddish-orange colour due to acridine orange binding to denatured DNA, were observed after 48 and 72 h treatment. Apoptotic cells undergoing secondary necrosis were also detected after 72 h of treatment. This provides qualitative evidence to proof that ZC-B11 induces apoptosis in treated CEMss cells. Acridine Orange (AO) and Propidium Iodide (PI) are intercalating nucleic acid-specific fluorochromes, which emit green and orange fluorescences, respectively, when bound to DNA. Only AO can cross the plasma membrane of viable and early apoptosis cells. This criterion of cell morphology identification according to the fluorescence colour density to distinguish apoptosis was previously reported by Ciapetti et al. [7]. 

### 3.3. ZC-B11 Induces Phosphatidylserine Externalisation in CEMss Cells

In this current investigation, PS externalisation of CEMss cells undergoing apoptosis was identified using Annexin V-FITC assay according to the manufacturer's instructions. The Annexin V-FITC assay apparently showed induction of early apoptosis in CEMss cells treated with ZC-B11 in a time-dependent manner (Table 2). The exposure time chosen for this experiment was 6, 12, 24, and 48 h for the purpose of an accurate detection of early apoptotic cells. For untreated control, 97.3% of cells were viable (Annexin V/negative; PI/negative) 2.2% of cells were in early apoptosis stage (Annexin V/positive; PI/negative), while 0.5% were in the late apoptosis (Annexin V/positive; PI/positive) and dead stage (Annexin V/negative; PI/positive). After 6 h of treatment, the viable cells decreased to 96.8%, while early apoptosis cells were only 3%. Viable cells decreased gradually to 95.2%, 86.7%, and 83.2% after 12, 24, and 48 h of incubation, respectively. On the other hand, early apoptosis cells increased from 3% at 6 h treatment to 3.8%, 12.8%, and 15.6% at 12, 24, and 48 h respectively (Table 2). The results obtained clearly indicate that ZC-B11 is able to induce apoptosis and simultaneously exhibit clear apoptosis morphological changes attributed to the induction of apoptosis reported previously using phase contrast inverted and confocal microscopy studies.

### 3.4. ZC-B11 Arrests the Cell Cycle at S-Phase and Induces Apoptosis

**Figure 4:**
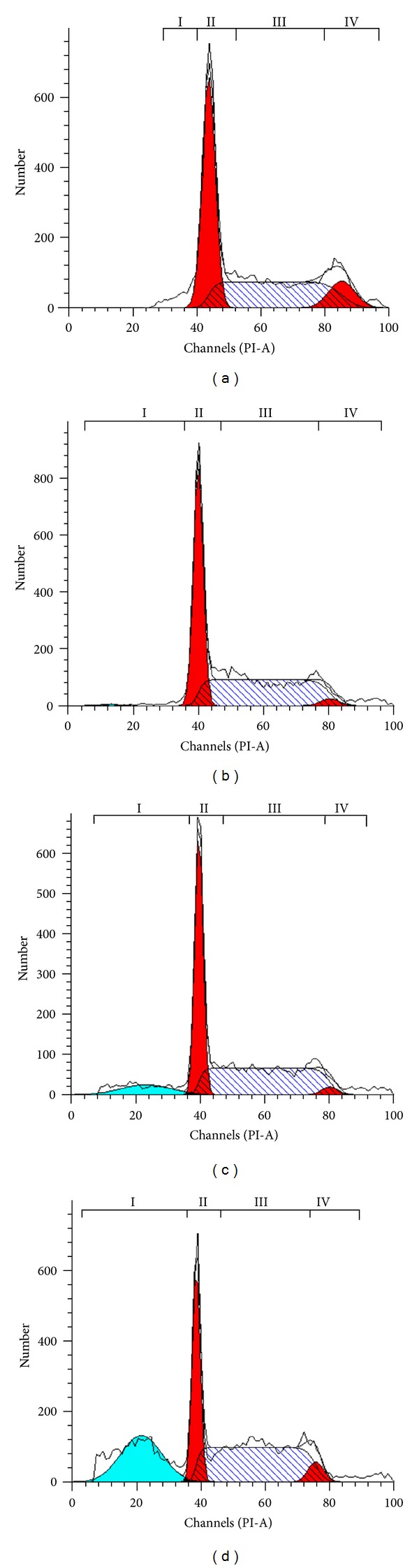
Flow cytometric analysis of cell cycle phase distribution of CEMss cells treated with ZC-B11 (IC_50_) in a time-dependent manner. (a) Control, (b) 24 h, (c) 48 h, and (d) 72 h. Region I is “sub-G0/G1” peak denoting apoptotic cells with hypodiploid DNA content, Region II is “G0/G1” phase, Region III is S phase, and Region IV is “G2/M” phase.

**Figure 5:**
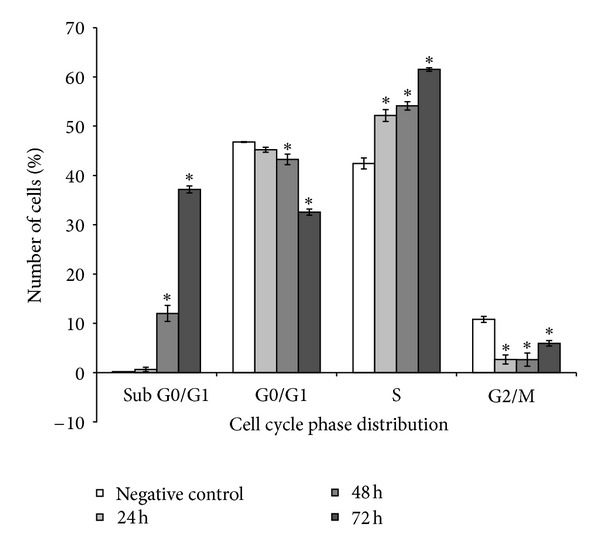
Graphical presentation of cell cycle phase distribution analysis. Induction of S phase arrest in the cell cycle progression of CEMss cells treated with ZC-B11 (IC_50_). Results were represented as means ± SD of three independent experiments. “∗” indicates a significant difference from the control (*P* < 0.05).

As depicted in Figures 4 and 5, there is a significant S phase arrest in a time-dependent manner, as the number of cells increased significantly from 42.43% (untreated control) to 52.16% after 24 h of treatment, followed by 54.13% and 61.51% for 48 and 72 h of treatment, respectively. The cells in sub-G1/G0 phase also increased significantly (*P* < 0.05) from 0.01% (untreated control) to 27.16% after 48 h of treatment. These cells are considered as apoptotic cells as the “sub-G1/G0” peak in DNA histogram denotes hypodiploid DNA content. Subsequently, the cells in the G0/G1 phase also decreased significantly from 46.78% (untreated control) to 43.25% and 32.55% after 48 and 72 h of treatment, respectively, promoting cell cycle arrest at S phase. 

### 3.5. ZC-B11 Triggers DNA Fragmentation Which Is the Hallmark of Apoptosis

**Figure 6:**
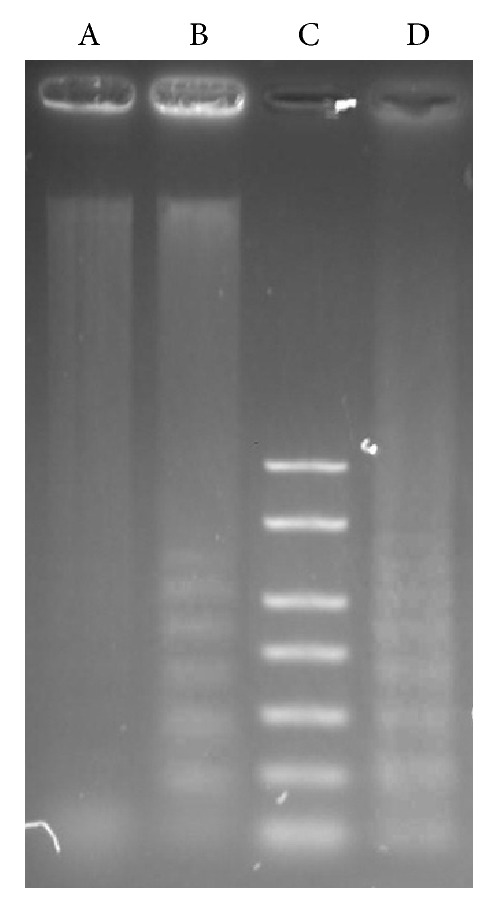
Electrophoresis separation of fragmented DNA of untreated and treated CEMss cells for 48 hours with ZC-B11 (IC_50_). Lane A: negative control (untreated CEMss cells); Lane B: 48 hours treatment; Lane C: DNA marker; Lane D: positive control.

In the current study, the formation of DNA fragmentation in CEMss cells treated at IC_50_ concentration of ZC-B11 was detected on a 1.2% agarose gel electrophoresis after 48 h treatment (Figure 6). Fragmented DNA was clearly observed in treated CEMss cells, whilst the untreated control did not show evidence of ladders. Thus, it is possible that the compound, ZC-B11, triggered apoptosis in CEMss cells as the chromosomal DNA cleavage into oligonucleosomal size fragments is an integral part of apoptosis induction.

### 3.6. ZC-B11 Causes Decline in Mitochondrial Membrane Potential

**Figure 7:**
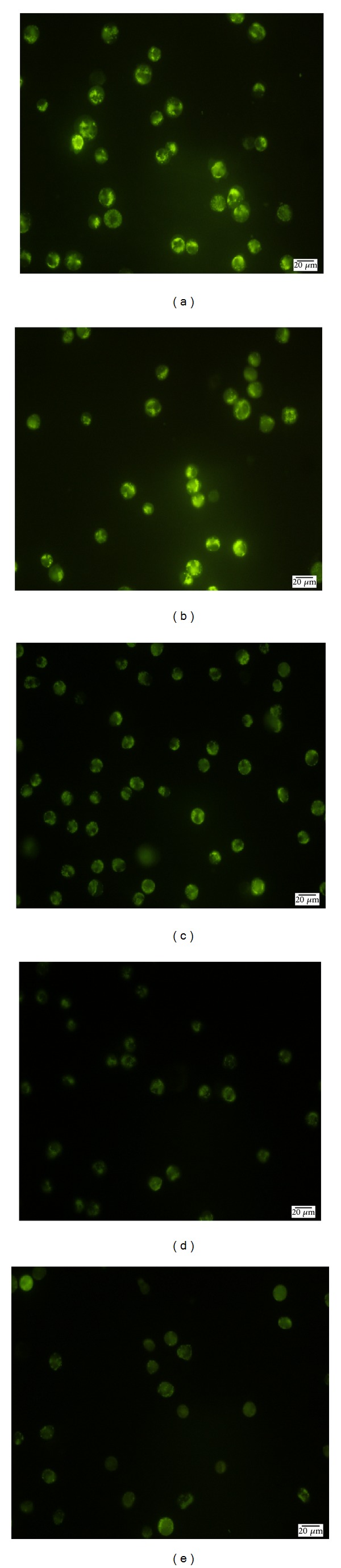
Fluorescent micrograph of CEMss cells treated with ZC-B11 (IC_50_) for 12, 24, 48 and 72 h, stained with Rh123 dye. (a) Control, (b) 12 h, (c) 24 h, (d) 48 h, and (e) 72 h. (400x magnification).

In the current study, mitochondrial membrane potential (MMP) was assessed by the retention of Rh123, a specific fluorescent cationic dye that is readily sequestered by active mitochondria [9, 10]. Uptake of Rh123 by CEMss cells treated with ZC-B11 was observed qualitatively using fluorescent microscopy. The fluorescent intensity of the Rh123 dye decreased sequentially in a time dependent manner from 12 h to 72 h of treatment, whilst untreated cells revealed maximal dye uptake (Figures 7(a)–7(e)). The result suggested that ZC-B11 disrupts the MMP of CEMss cells after treatment.

### 3.7. ZC-B11 Upregulates Bax, Caspase 3, Cytochrome c, and SMAC, Downregulates Bcl-2, HSP70, and XIAP but Did Not Affect Caspase 8, p53, and BID

**Figure 8:**
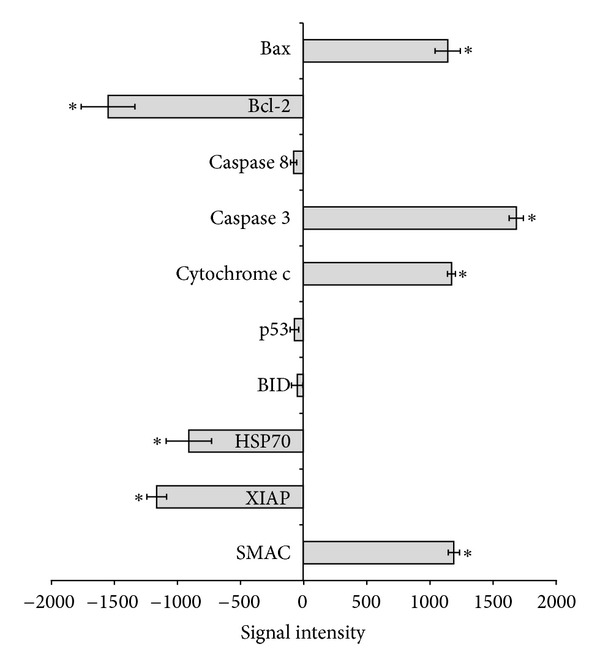
Human apoptosis proteome profiler array in CEMss cells treated with ZC-B11 (IC_50_) for 48 hours. Graph shows the difference between treated and untreated control cells. Results were represented as means ± SD for three independent experiments. “*” indicates a significant difference from the control (*P* < 0.05).

To further evaluate the mechanisms of apoptosis induction by ZC-B11 towards CEMss cells, screening of several proteins implicated to apoptosis induction was done using the human apoptosis proteome profiler array. Bax, caspase 3, cytochrome c, and SMAC showed significant increase (*P* < 0.05) compared to untreated control cells, whilst proteins such as Bcl-2, HSP70, and XIAP decreased significantly compared to untreated control cells (Figure 8). On the other hand, caspase 8, p53, and BID do not show significant difference from untreated control cells. The upregulation of Bax, cytochrome c, caspase 3 and the downregulation of Bcl-2 suggest that the compound induces apoptosis in CEMss via intrinsic pathway. This is further confirmed with unchanged levels of caspase 8 and BID, which plays important role in extrinsic pathway of apoptosis. Induction of apoptosis is independent of p53 as the expression of this protein remained at basal level after treatment. Increased levels of cytochrome c and SMAC correlate well with the decline of mitochondrial membrane potential as mentioned earlier. An alteration in the permeability of mitochondrial membranes promotes translocation of the mitochondrial apoptogenic proteins which included SMAC (an inhibitor of XIAP) and cytochrome c (an activator of caspase 9)into the cytoplasm. Interestingly, there was significant decline in the level of XIAP (apoptosis inhibitor protein) suggesting possible inhibition of this protein by increased level of SMAC. Hence, the evidence gathered by the present study proposed that SMAC acts as a proapoptotic protein that binds and neutralizes the activity of XIAP. Alternatively, the level of HSP70 showed significant decrease compared to the untreated control. HSP70 is an inhibitor of apoptosis since cellular-stress response can mediate cellular protection through the expression of HSP70, which in turn can interfere with the induction of apoptotic cell death, hence resulting in tumour cells often expressing elevated levels of HSP70 [11, 12]. A previous study has found that HSP70 inhibits apoptosis downstream of cytochrome c release and upstream of caspase3 activation [13]. Hence, in the current study, treatment of ZC-B11 towards CEMss cells decreases HSP70 protein activity, thus preventing its inhibition on cytochrome c release and caspase 3 activation. The current findings from the apoptosis proteome profiler array suggest that ZC-B11 induces apoptosis in CEMss cells which is independent of p53 protein expression and may not involve the extrinsic pathway. Further to this, ZC-B11 may possibly activate the caspase cascades in CEMss cells, accompanied by the release of SMAC and the subsequent suppression of XIAP and HSP70 proteins.

### 3.8. ZC-B11 Increases the Activity of Caspases Involved in Intrinsic Pathway

**Figure 9:**
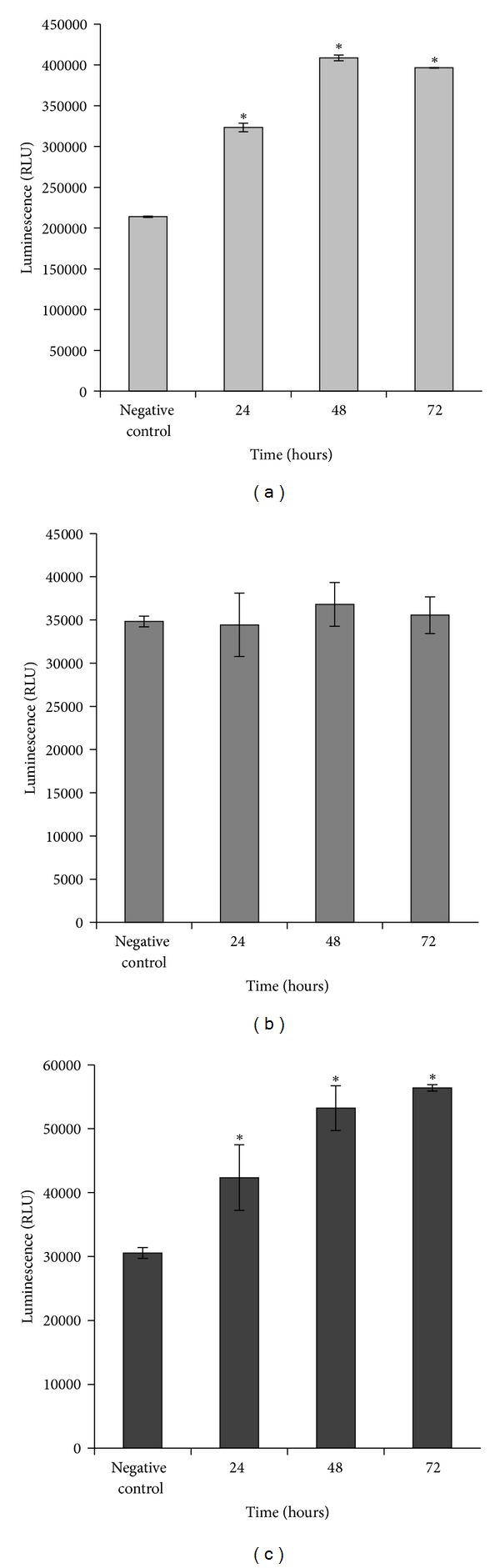
The bioluminescent assay of caspases 3/7 (a), 8 (b), and 9 (c) in CEMss cells treated with ZC-B11 (IC_50_) after 24, 48, and 72 hours of treatment. Untreated cells serve as negative control. (a), (c) Caspase 3/7 and 9 activities increased significantly (*P < 0.05) compared to untreated control; (b) Caspase 8 activity remained at the basal level throughout the treatment period. Results were represented as means ± SD for three independent experiments.

CEMss cells treated with IC_50_ concentration of ZC-B11 significantly (*P* < 0.05) increased the activities of caspase 3/7 (Figures 9(a) and 9(c)) in a time dependent manner whilst the activity of caspase 8 (Figure 9(b)) remained unchanged throughout the treatment period. Caspases 3/7 and 9 are caspases that are involved in the intrinsic pathway and this strongly suggests that ZC-B11 induces apoptosis in CEMss cells via intrinsic pathway. The formation of apoptosome, a catalytic multiprotein complex consisting of Apaf-1, cytochrome c, and procaspase 9 within the intrinsic apoptotic pathway, activates caspase 9 in response to the apoptotic signals, which contributes later to the activation of caspase 3 [14, 15]. In relation to this,the current study suggests the possibility of ZC-B11 inducing the formation of apoptosome complex in treated CEMss cells, since this protein complex is implicated in activating both caspases 3 and 9 of the intrinsic pathway.

### 3.9. Western Blotting Confirms the upregulation of Bax and downregulation of Bcl-2, HSP70 Induced by ZC-B11

**Figure 10:**
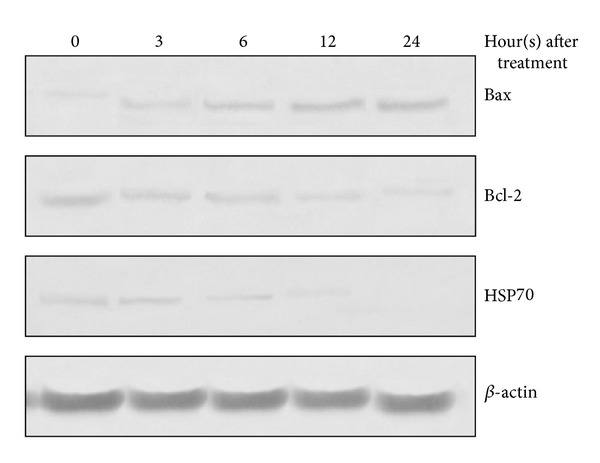
Western blot analysis of Bax, Bcl-2, and HSP70 levels in CEMss cells treated with ZC-B11 (IC_50_) for 3, 6, 12, and 24 hours and compared with negative control (0 hours). The expression of Bax increased while Bcl-2 and HSP70 decreased after treatment in a time-dependent manner. *β*-actin was used as the internal control to confirm equal sample loading.

ZC-B11 increased the expression of Bax while the expression of Bcl-2 and HSP70 decreased after treatment in a time-dependent manner compared to untreated control cells. *β*-Actin was used as the internal control to confirm equal sample loading and protein concentration in all samples. The results obtained from the Western blot analysis confirmed that ZC-B11 induced up regulation of Bax and down regulation of Bcl-2 and HSP70 proteins in a time dependent manner (Figure 10) and these were concurrent with previous human apoptosis proteome profiler array results. As Bax and Bcl-2 are the main orchestrators of apoptosis regulation, the ability to upregulate and downregulate these proteins optimally to induce apoptosis is a crucial attribute for an anticancer agent, and ZC-B11 has demonstrated this capability when used to treat CEMss cells.

## 4. Discussion and Conclusion

Over the last decade, countless studies have revealed that the response to current cancer therapies crucially depends on functional cell death pathways in cancer cells. Recognition of key regulators of apoptosis in childhood cancers has provided the basis for the advancement of experimental strategies aiming at restoring intact cell death programs in cancer cells [16]. The present study elucidates the mechanism of apoptosis provoked by ZC-B11 in CEMss cells. 

The antiproliferative assay used in this study is the MTT assay, an *in vitro *tetrazolium-based colorimetric assay. The method was first described by Mosmann in 1983 for detecting mammalian cell survival and proliferation [17]. MTT (3-[4,5-dimethylthiazol-2-yl]-2,5-diphenyltetrazolium bromide) is a water soluble tetrazolium salt, which is reduced to an insoluble formazan product by cleavage of the tetrazolium ring by succinate dehydrogenase enzyme within the mitochondria of metabolically active cells [18]. The amount of formazan generated is directly proportional to the viable cell number when using homogenous cell populations. This technique has since been accepted largely for its accuracy and speediness in quantifying cell survival and proliferation [19, 20]. It is well acknowledged that different cell lines demonstrate different sensitivities to cytotoxic compounds [21, 22]. The use of more than one cell line is therefore necessary in screening the antiproliferative activity of ZC-B11. The five different cancer cell lines used are from four different origins (blood, breast, liver, and cervix) that possess different morphology and tumorigenic properties [22]. The data obtained in this experiment demonstrated the antiproliferative effects of ZC-B11 on CEMss cell line selectively without affecting the normal blood mononuclear cells.

The antileukemic activities of ZC-B11 were further established using various microscopic analyses and AO/PI staining, which showed distinctive morphological changes corresponding to typical apoptosis features such as chromatin condensation, DNA fragmentation, cell membrane blebbing, and separated apoptotic bodies. The inclusion of PS externalization study and DNA fragmentation analysis further confirmed the induction of apoptosis as the event of causing cell death towards CEMss cells. Annexin V is a 35-kDa Ca^2+^-binding protein initially described by Reutelingsperger et al. (1985) as a vasculature-derived protein with strong anticoagulant properties [23]. Annexin V binds preferentially to PS, which is normally absent in the outer leaflet of the plasma membrane and is only exposed on the cell surface upon induction of apoptosis. Once on the cell surface, it can be specifically detected by staining with fluorescein isothiocyanate-(FITC-) labeled annexin V (annexin V-FITC), a protein with high affinity for PS. This occurs specifically in early phases of apoptotic cell death during which the cell membrane itself remains intact [24, 25]. Fragmentation of DNA also appears to be part of an early event in apoptosis, prior to the complete digestion of DNA into multiples of nucleosomal size fragments. Demonstration of internucleosomal DNA fragmentation has been the major advance in the detection of apoptosis induction [26]. DNA fragments are detectable as a ladder pattern in gel electrophoresis of isolated DNA. Necrosis, on the other hand, is characterized by accidental DNA fragmentation which forms a “smear” on agarose gels [27]. 

ZC-B11 also causes DNA damage in CEMss cells by arresting the cell cycle at S phase. This is in accordance to several studies elsewhere that reported compounds isolated from natural products which arrested cell cycle at S phase similarly induce apoptosis. Triptolide, a diterpenoid obtained from *Tripterygum wilfordii* Hook. if was reported to induce S phase cell cycle arrest in human melanoma cells and apoptosis that probably is induced through the intrinsic pathway [28]. Resveratrol, a polyphenolic phytoalexin found in the skin of red grapes, various other fruits, and root extracts of the weed *Polygonum cuspidatum,* also inhibits proliferation, causes S-phase arrest, and induces apoptosis in acute myeloid leukemia and several other cancer cells [29, 30]. 

Mitochondria are the main producers of ATP in eukaryotic aerobic cells. In addition, mitochondria also implicated cell physiology and pathology, including involvement in ions homeostasis, regulation of the cell redox state, and transport of metabolites, including import of proteins synthesized in the cytosol, lipid and amino acid metabolism, and cell death. These important functions are extremely dependent on the electrochemical transmembrane potential of the mitochondria [31, 32]. The disruption of mitochondrial membrane integrity is one of the early events leading towards irreversible apoptosis [33]. Mitochondrial inner membrane is negatively charged for being rich of negatively charged glycoprotein. A large migration of protons out of the inner membrane causes the transmembrane potential to decrease substantially [34]. Decline of MMP causes leakage of Rh123 from mitochondria, resulting in the decline of fluorescence intensity [35]. The rate of fluorescence decay is proportional to the decline of MMP [34]. The decline of MMP in ZC-B11-treated CEMss cells was also found to be concomitant with the upregulation of cytochrome c and SMAC, which are the key apoptotic proteins from mitochondrial intermembrane space. Therefore, one possible mechanism by which ZC-B11 induces apoptosis is through changes in the MMP, which would lead to the release of cytochrome c and SMAC from the mitochondria, leading to sequential activation of caspase 9 and caspase 3. Furthermore, regulation of several apoptosis-related proteins was also observed in the human proteome profiler array, which suggest collectively that ZC-B11 mediates cell death in CEMss via intrinsic pathway of apoptosis. These results were later confirmed by caspase bioluminescent assay and western blot analysis. HSP70 which shows decline in ZC-B11-treated CEMss cells is a protein that is abnormally expressed in different malignancies and has emerged as a promising new target for anticancer therapy [36]. The expression of p53 was found to be unaffected in ZC-B11-treated CEMss cells which suggest a probable p53-independent pathway of apoptosis. Since over 50% of human tumours contain a functionally defective p53 that reduces sensitivity to commonly used chemotherapeutic agents, such as etoposide and cisplatin [37], the aptitude of ZC-B11 to induce apoptosis independently of p53 may offer an advantage in anticancer therapy. Ultimately, this study has shown that ZC-B11 possesses antiproliferative properties on acute lymphoblastic leukemia and is potential to be developed as an antileukemic and chemotherapy agent as it induces cell death via apoptosis signalling pathway.

However, *in vivo* preclinical studies and possibly further product development are needed to ascertain its ability and usefulness as a natural drug for the treatment of leukemia. Direct engagements of apoptotic pathways or combinations of agents that lower the threshold for apoptosis induction by conventional anticancer agents are alternatives to exploit apoptosis induction pathways for paediatric oncology such as ALL [16]. These findings will therefore help in understanding the mechanism by which apoptosis pathway is regulated in ZC-B11-treated ALL and consequently provides valuable links for developing strategies to improve the efficacy of anticancer therapy.

## Figures and Tables

**Table 1:** Effect of ZC-B11 on different cell types and the effect of 5-fluorouracil on CEMss cell line expressed as IC_50 _values in MTT assay after 72 hours. ZC-B11 potently inhibits the growth of T-acute lymphoblastic leukemic cells.

Cell line	Tissue of human origin	Compound IC_50_ (*μ*g/mL)	
		ZC-B11	5-Fluorouracil
CEMss	T-Acute lymphoblastic leukemia	7.11 ± 0.240	1.54 ± 0.035
MCF-7	Human breast adenocarcinoma	21.28 ± 0.251	—
MDA-MB-231	Human breast carcinoma	32.38 ± 0.412	—
HepG2	Hepatocellular carcinoma	17.65 ± 0.323	—
HeLa	Cervical carcinoma	>50	—
—	Human blood mononuclear cells	>50	—

**Table 2:** Flow cytometric analysis of Annexin V:FITC assay in CEMss cells treated with IC_50 _concentration of ZC-B11 for 6, 12, 24, and 48 hours. Untreated cells serve as negative control. Data were represented as means ± SD of at least three independent experiments.

	Number of cells (%) ± SD				
	Untreated	6	12	24	48
Viable	97.30 ± 0.42	96.80 ± 0.35	95.20 ± 0.57	86.70 ± 1.06	83.20 ± 0.78
Early apoptosis	2.20 ± 0.21	3.00 ± 0.14	3.80 ± 0.42	12.80 ± 1.13*	15.60 ± 1.20*
Late apoptosis/secondary necrosis	0.50 ± 0.21	0.30 ± 0.49	0.60 ± 0.07	1.10 ± 0.21	1.20 ± 0.43

*indicates a significant difference from the control (*P* < 0.05).

## Abbreviations

ALL: Acute lymphoblastic leukemia
Apaf-1: Apoptotic protease-activating factor-1
Bax: Bcl-2-associated X protein
Bcl-2: B-cell lymphoma 2
BID: BH3 interacting domain death agonist
DMSO: Dimethyl sulfoxide
EI-MS: Electron impact-mass spectra
EtOAc: Ethyl acetate
FITC: Fluorescein isothiocyanate
HPLC: High-performance liquid chromatography
HSP70: Heat shock protein 70
IC_50_: Half-maximal inhibitory concentration
IR: Infrared
MMP: Mitochondrial membrane potential
MTT: 3-(4,5-Dimethylthiazol-2-yl)-2,5-diphenyltetrazolium bromide
NMR: Nuclear magnetic resonance
SMAC: Second mitochondria-derived activator of caspase
XIAP: X-Linked inhibitor of apoptosis protein.
